# Mental Health and Hospital Chaplaincy: Strategies of Self-Protection (Case Study: Toronto, Canada)

**Published:** 2013

**Authors:** Masoud Kianpour

**Affiliations:** 1 Assistant Professor of Sociology, Department of the Social Sciences, Isfahan University, Iran. Department of the Social Sciences, Faculty of Literature and the Humanities, Isfahan University, Iran.

**Keywords:** Emotion Management, Hospital Chaplaincy, Mental Health

## Abstract

**Objective:** This is a study about emotion management among a category of healthcare professional – hospital chaplains – who have hardly been the subject of sociological research about emotions. The aim of the study was to understand how chaplains manage their work-related emotions in order to protect their mental health, whilst also providing spiritual care.

**Methods:** Using in-depth, semi structured interviews, the author spoke with 21 chaplains from five faith traditions (Christianity, Islam, Judaism, Buddhism and modern paganism) in different Toronto (Canada) Hospitals to see how they manage their emotion, and what resources they rely on in order to protect their mental health. Data analysis was perfumed according to Sandelowski’s method of qualitative description.

**Results:** The average age and work experience of the subjects interviewed in this study are 52 and 9.6 respectively. 11 chaplains worked part-time and 10 chaplains worked full-time. 18 respondents were women and the sample incudes 3 male chaplains only. The findings are discussed, among others, according to the following themes: work-life balance, self-reflexivity, methods of self-care, and chaplains’ emotional make-up.

**Conclusion:** Emotion management per se is not a problem. However, if chaplains fail to maintain a proper work-life balance, job pressure can be harmful. As a strategy, many chaplains work part-time. As a supportive means, an overwhelming number of chaplains regularly benefit from psychotherapy and/or spiritual guidance.

Declaration of interest: None.

## Introduction

As medicine began to expand at an unprecedented rate from the early 19th century in Western, industrialized societies, religiously charged institutions that were traditionally responsible for providing medical service were gradually replaced by professional, more secular, medical institutions.While medicine has become more efficient and effective, at the same time it has developed an impersonal approach toward the patient, viewing him or her as a machine.Physicians are criticizedfor losing the kind of compassion and empathy with which they used to treat patients. They are taught to become emotionally detached and objective (1). Consequently, modern physicians have less time to spend with individual patients and their previous roles as attentive healers are changed as they become highly educated technicians, whose relationships with patients are contractual (2).

In response to the domination of modern medicine, which seems to be highly technical but lacking sensitivity, hospital chaplaincy gained momentum. After all, religion can influence health in both positive and negative ways. For example, being angry with God after a terminal diagnosis, or an inability to make sense of intense suffering, can further intensify a health problem.On the other hand, strong faith can help people cope with sickness and pain (3, 4). Hospital chaplaincy provides support for patient’s religiosity and spirituality, identifying emotional problems related to religious values and tries to reconcile them.

As an integral part of the healthcare system in North America, hospital chaplains have become increasingly professionalized (5), meaning that they can be trained and employed by hospitals in full-time jobs as spiritual care providers. In 1969, the Joint Commission for the Accreditation of Healthcare Organizations (JCAHO) in the United States provided guidelines for religion and spirituality in hospitals for the first time. In 2003, JCAHO declared that medical services should be offered in such a way that patients’ personal, cultural, psychological, and spiritual values would be respected. As a consequence, hospitals are required to demonstrate respect for patients’ needs, including the need for pastoral care and spiritual support. According to a survey by Cadge and colleagues (6), between 54% and 64% of hospitals had chaplaincyservices between 1980 and 2003in the Unites States. It is estimated that more than 10,000 professional chaplains work in American hospitals. 

Today, hospital chaplains deal with a wide range of issues including religious and spiritual values, dietary options, pain concerns, end-of-life issues, and the treatment and responsibilities of medical staff. Their daily work may include providing “emotional, practical, ritual and crisis intervention services to patients, families, and staff individually or as members of health care teams (7-9).

There are several studies on the emotion management of different healthcare professionals (10).For example, by arguing that individuals can use either a behavioral mode or a cognitive one to alter their emotions, Thoits (11) has created an eight-fold classification of emotion management techniques including, among others, leaving the situation, catharsis, taking direct action, seeking support, hiding feelings, and seeing the situation differently. However, hospital chaplaincy is a rather recently professionalized occupation which has been little studied (12-14).As employed and paid members of the hospital in which they work, the chaplains’ role is organizationally established like other members of the healthcare system. Therefore, they may well be subject to specific feeling rules (15) and expectations with regard to the management and expression of emotions. This study tries to understand how chaplains manage their emotions in order to beable to perform their job successfully. In doing so, a qualitative approach has been used to provide a deeper understanding of the subtleties of emotion management. Qualitative research has two important features. First, it can be useful for explorations in areas about which little is known (16, 17). Second, it can provide novel and fresh understanding, in detail, of phenomena that are difficult to examine with quantitative methods (18).

## Materials and Methods

This study is based on a primary data source of 21 in-depth interviews with professional chaplains working, full-time and part-time, in different Toronto Hospitals. 

As one of the largest cities of Canada, Toronto has more than 30 hospitals and medical centers, most of which have a spiritual care or chaplaincy department. By estimation, 40 to 50 professional chaplains work across Toronto hospitals. Therefore purposeful sampling, a non-random method of sampling in which the researcher selects “information-rich” cases for in-depth interview (19), was used to address all potential respondents across different hospitals in a direct and straightforward manner, asking for their voluntary participation. All hospital chaplains who had work experience were potentially “information-rich” cases. In cases where it was mandatory, ethics acceptance was obtained from Research Ethics Boards. 

Sampling continued until theoretical saturation, namely, where no new conceptual patterns were emerging from new interviews. Research questions were predominantly related to chaplains’ work-related emotional experiences, the resources they rely on in order to protect their mental health and strategies they use while doing their job. 

Qualitative fieldwork may be characterized as emergent and interactive. As Loftland and Loftland (20) argue, gathering, transcribing, coding and analyzing data are complex, overlapping and interweaving phases of the research process. In this research all interviews were transcribed verbatim. As a non-native speaker of English, the researcher faced an additional challenge of making sure that words and sentences were as accurate as possible. Fortunately, most respondents tended to speak slowly and with good articulation. As interview transcripts were critically examined line by line, the researcher was attentive towards what was being revealed. The insights that were developed from the preliminary analysis shaped subsequent stages of data collection. Moreover, the researcher tried to approach each transcript, as Miles and Huberman (21) say, “prepared to let the interview breathe and speak for itself.” In order to analyze the data, Sandelowski’s(22) method of qualitative description was used, with a phenomenological inclination, that is, the goal was to describe emotion management experiences as they are lived and felt by respondents.

Qualitative description is a method of analyzing data that researcherscan claim unashamedly without resorting tomethodological acrobatics.Such qualitative analysis is the least interpretive of the different qualitativeanalysis approaches (e.g. phenomenological, ethnographic, grounded theory, etc.), in that there is no mandate tore-present the data in any other terms but their own.

## Results

Chaplains who participated in this study were between the ages of 33 and 65. The average age is approximately 52. The least experienced chaplain had two and half years of work experience, in contrast to a chaplain who started her career 26 years ago and was retired from her full-time position but was still working part-time. The average work experience in the sample is 9.6 years. Also, 11 chaplains worked part-time and 10 chaplains worked full-time. 18 of the 21 chaplains in this sample are women. Recruiting more than 3 male chaplains was not possible due to the fact that hospital chaplaincy is a job predominantly occupied by women. In terms of ethnicity, the majority of the respondents are white, with European and Anglo-Saxon backgrounds. However, the sample also includes two Asian chaplains (with Chinese and Indian backgrounds) and one from the Caribbean Islands. 

In terms of religious affiliation, the sample includes chaplains from five different religions and faith traditions. The majority of the chaplains were Christian, including five chaplains belonging to the Anglican Church, three to the Roman Catholic Church, two to the United Church of Canada, and one to the Baptist Church. The remaining four Christian chaplains did not specify their Church. Several of the chaplains were church ministers. Also, two chaplains from Buddhism, one from Islam, and one from Judaism were interviewed. Finally, a pagan chaplain, who is a believer in the modern paganism movement and is apparently one of only two such chaplains in Canada, was interviewed. 

***Strategies of Emotion management***

*1. Work-Life Balance: Emotional Separation*

All participants of this study were able to identify several sources of support they can benefit from at different points, separately or together, in order to protect their health. There are also various strategies of emotion management and methods of self-care that enable them to avoid or mitigate occupational risks. For example, In order to maintain a work-life balance, chaplains consciously manage to separate work from other aspects of their lives. Like other healthcare professionals, they seek to obtain some degree of objectivity in order to put concrete boundaries between work responsibilities and personal life. Several chaplains described specific rituals as strategies they use in order to separate the work environment from home life. 

*My symbolic thing, every day, is to transfer my pager to the person who is on-call and knowing that that’s it: I am no longer responsible for anybody here at the end of the day, you know; I would pick it up tomorrow. It’s my way of….work is over. It’s almost like an emotional break from the place; just transferring my pager to the person who is on-call and to say: “it’s all yours now” (laughter) *(Interview No. 13).

As a cognitive method of emotion management (15), the following chaplain performs her job as effectively as she can and leaves the rest in “God’s hands”: 

*My attitude is when I am here I am gonna do the best job that I know how and when I leave, I leave it in God’s hands and so I don’t have to worry about people not being well afterwards, because I know God is gonna look after them. I am not God, God is always gonna be here so I can go at the end of day, you know, knowing that I leave them in good hands and the rest will be still here when I come back. A friend of mine always says: “why did God make tomorrow?” So we can come back and pick up what we left off *(Interview No. 12).

Similarly, another chaplain talks about his daily practice to separate the hospital and home environments: 

*I walk to work. It takes me a half hour to walk to work and then a half hour to walk home, and for me the process of coming here…because I am not rushing with the subway or driving….I don’t have to pay a lot of attention to my route... I am just walking…preparing myself emotionally to come into this environment and be spiritual care provider here and on the trip to home to divest myself of that, sort of free myself of the experience of being here which is very much…the normal feelings in the hospital are sense of ambiguity, of not knowing, of fear, of anxiety, and to sort of divest myself of that on my walk back home, I think is a really important part of my personal process. It’s a walking meditation… *(Interview No. 8).

Similarly, another chaplain explains: 

*I try to consciously wash my hands before I go in as a way of preparing myself for when I walk into the space, and then when I leave, the washing of my hands is also a way of leaving it behind so that I don't carry it home… *(Interview No. 14).

Such idiosyncratic practices can be described at ritualistic methods of emotion management. Since chaplains perform these rituals on a daily basis, they can be also described as daily rites of passage. Studied frequently by anthropologists, a rite of passage is a ritual that shows what social hierarchies, values and beliefs are important in specific cultures. Rites of passage are often ceremonies surrounding important events such as puberty, coming of age, marriage and death (23). Since practices discussed here are personal, contextual, and idiosyncratic, they can be described as micro, daily, rites of passage used to manage emotions. 

By using such rituals, chaplains unconsciously separate their work from other areas of life, keeping in mind that what happens in the hospital should not be seen as their personal pain, problem or hardship. Although once in a while it may become difficult to get over a particular case, in most cases chaplains are able to move forward and put their work-related thoughts behind them. They should be able to separate other people’s pain and suffering from their own or they fail to survive in this job. As one chaplain said:

*I join them [patients] and travel with them but in the same token there is a certain amount of professional distance that I have because at the end of the day this isn’t my family, this isn’t my best friend; we go our separate ways at the end of our time together and I don’t miss these people at birthdays and family gatherings *(Interview No. 15).

As another part of maintaining work-life balance, many chaplains decide to work part-time so that they do not have to spend more than two-and-half days in hospital, or commute long distances. One chaplain decided to work part time to avoid driving long distance on a daily basis from home to work and vice versa: 

*I used to work full-time for a contract position on oncology and palliative care, so I know what the burnout rate at this profession is, but I learned pretty quickly that with the pace I was going: working full-time and commuting…I have a long commute that did not give me enough solitude to recharge and to have that balance, so I chose to work half-time so that I can still have a family life, my professional life and some time for me to recharge… that’s very intentional; I couldn’t do that and even now sometimes I struggle because there are many demands and it can be hard to balance everything *(Interview No. 15). 

*2. Self-reflexivity*

Hospital chaplaincy is very reflexive work. Chaplains must be very familiar with their own emotional reactions to different situations. After all, successful emotion management is only possible when a chaplain knows what his or her limitations and triggers are. This is a point stressed by several respondents. One experienced chaplain talked about brownout as an indicator that comes before burnout:

*We have to watch for brownout, and brownout is when you are in a city and the lights dim but they don’t completely go out and then sometime later they do, and sometimes later they come back on, so that’s what I monitor myself for all the time: brownout. If I feel getting dim, then I take measures… *(Interview No. 12).

In their professional training, chaplains are vigilant about identifying and naming their own emotions so that they can effectively manage them. For example, there are situations where chaplains feel angry over medical personnel but suppress or try to transform their feelings into something milder. Mindful of the potential harms such emotions may bring, chaplains remind themselves to let them go. Chaplains may actually talk themselves into changing a feeling or recognizing it as something different. One respondent described a mental mechanism she benefits from whenever emotions are high in a situation:

I have a natural, for better or worse, shut down mechanism (laughter), you might have been hearing about this from other folks as well; so I am monitoring what I do – it’s like watching my feelings, you know: “Amélie, what’s happening with you?” and then if…it can…it might even happen not as a conscious choice, there were times when it didn’t, it wasn’t a conscious choice, there would be a process of numbing out those feelings and*becoming very heady, so my rational mind would take over: “Amélie, what do you need to do to manage the situation?” So my rational mind would say [to my emotional mind] like: “you sit in the back, I am taking over here;” and, you know, it would be all about what needs to be done *(interview No. 19). 

Such self-talks or self-reminders are psychological methods of emotion management that chaplains use whenever they find themselves in a stressful or crisis situation. They can be explained by Denzin’s (24) phenomenological approach in which emotions are seen as products of the social interplay between inter and intra personal interactions. According to Denzin, our emotionality is influenced by inhibited social acts, sub vocal thought, interpretations and self-conversations in social action.

*3. Methods of Self-Care: Physical, Spiritual and Emotional Well-being*

In addition to the psychological mechanisms, and specific rituals discussed above, chaplains need to be extremely attentive to their self-care and well-being so that they can maintain a high level of health. Participants of this study were able to articulate a variety of different methods of self-care and strategies to cope with emotional difficulties. All respondents showed a heightened level of attentiveness to their spiritual life and emotional well-being. Prayer, meditation, reflection, listening to music, exercise, balanced diet, sufficient sleep, participation in life-giving activities, socializing with friends, community work , silent retreats, vacation trips, professional training, and educational workshops are the typical means through which chaplains maintain the well-being needed to perform their job. They can use a combination of different mechanisms at the same time or, depending on the situation, one particular method may be given priority over the others. When it comes to self-care,taking good physical care of oneself is extremely important. Obviously, this job requires full commitment and full emotional presence. Chaplains must be attentive to their own needs so that they can perform well on the job. Speaking from a holistic perspective, one chaplain said that in order to be emotionally stable, her physical needs must be met first: 

*“Usually I have an intense day and often I go to the gym and I work out; I am very…I eat really well, I take very good care of my diet and try balance it so I don’t eat processed food or a lot of processed food…I am very careful about that….I make sure that I get a good sleep; I meditate on a daily basis… so those are the sort of the things that I do….” *(Interview No. 6).

The spiritual life of chaplains, in particular, is very important to give them a sense of orientation and groundedness. Spiritual life can be enriched through, among other things, prayer and meditation. For several chaplains, prayer was an indispensable part of their daily work. One chaplain said: *“I do pray and ask for God’s guidance and wisdom before I go to a situation” *(interview No. 9). For the only Muslim chaplain in the sample, it was important to maintain his Salat. Another chaplain said: 

*I pray every day. By giving the situation to the lord, praying that I would have the strength to go back to the next room and be as compassionate with the next person as I was with the first person. That is why my prayer list is so long, because I pray for a lot of people and I still feel that they are in larger hands than mine… *(Interview No. 17).

“Giving the situation to the lord” is a cognitive technique of emotion management. Coupled with prayer as a ritualistic technique, it helps this chaplain perform her job. Some chaplains distinguished between prayer and meditation. Even though several chaplains said they meditate regularly and separated this from prayer, meditation seemed to be specifically significant in the words of the two Buddhist chaplains, whose religiosity is more embedded in meditative practices. 

Among other chaplains, one talked about long walks as a therapeutic, meditative exercise to allow *for “clearing my head and having time to reflect on myself and my own spiritual life” *(interview No. 13). Another chaplain talked about music as an important outlet:

*“ I love music, and on the way over I almost always listen to…I like classical music best and I always find music as one of God’s gifts. I really feel, you know, I don’t know; when you think about it, that we are created in such a way that somebody has the gift of composing the music and another person has the gift of playing the music and that our bodies are made in such a way that we hear sounds and it does something to our souls, to our bodies; I found that miraculous, God’s work, so I always listen to music and I find that very helpful and that helps me get some sort of nice frame of mind” *(Interview No. 21).

Several respondents talked about entertaining, life-giving activities such as going to an art gallery, or doing “creative” and “beautiful” things as methods of self-care which help battle work pressures. Sometimes they may do nothing important and let time pass. As one chaplain said: *“sometimes I watch TV and if I choose to watch TV, it is completely mindless stuff” *(interview No. 12).Another chaplain used a cognitive technique to cope with sadness: as a result of working in hospital, she has developed *“a rapidly decreasing tolerance for exposure to suffering outside of work.”* Consequently, she does not like to listen to the news or read the newspaper when it is full of pain and suffering. As she puts it: *“I found that I need to limit my exposure to sad things outside of hospital” *(interview No. 18). 

 Also, a few respondents were more intellectually involved in reading and writing short stories, poetry, prayers or journaling as outlets to deal with their feelings. One of the Buddhist chaplains was co-authorof a book about traditional Chinese medicine and women’s health. 

Apart from the above practices, some chaplains benefit from silent retreats. Described as a *“vacation with God”* (interview No. 12) by one of the respondents, such retreats are usually characterized by a short, intentional period of isolation in which chaplains disconnect themselves from the outside world, and stay in quiet places with no TV or cell phone use. This temporary withdrawal from the routine, highly industrialized and commercialized world provides chaplains with an opportunity to reflect on their inner lives and attend to their own spiritual needs. 

As another source of support, chaplains participate in various educational workshops, occupational training courses or meetings with other colleagues to not only reinforce their institutional role, but also to reflect on the job and receive insight. Such meetings and workshops provide what can be called self-presentational strategiesof interaction to make connections with colleagues:

Our chaplains meet once a week for something called Dialogue and we begin with the reflection; we take turns and each person has a different way of doing it. They bring over reflections which lead into some discussion about that, which leads to a staff meeting. Yesterday, for example, the chaplain who led the Dialogue, she brought something on wonder; she brought an experience she had with her granddaughter and then read something from the book on wonder and then we talked about that....*so that's another way of coping with our, you know, the situations we have because sometimes we can't bring to that meeting… if we’ve got a patient we are concerned about. If you have got a woman upstairs who is just losing her baby, you know, a 25-week pregnant, that's one of the hardest things to deal with. So being able to talk to others, about it, about how it's affecting you...* (Interview No. 4). 

*4. Chaplains’ Emotional Make-up*

Chaplains develop a different perspective towards their job. For many people, hospitals are synonymous with bad news. Most frequently associated with blood, white uniforms, medical instruments, pain, suffering, illness, and ultimately death, hospital is a place to be avoided as much as possible. Chaplains, however, tend to look at these notions differently. They do not enjoy illness and death but gain satisfaction from helping people who are struggling with them. Even though the job is often full of sad stories, and even though situations are stressful, chaplains feel emotionally rewarded because their emotional make up has developed in such a way that they gain pleasure (not in a hedonistic way but in a fulfilling way) from being in the presence of troubled people as care providers, helping them go through their hospital journey. As one respondent suggests:

*“Knowing that you have made a difference in someone’s life is energizing, so some days I come away from a challenging situation knowing that I have done a good job, knowing that I have made a difference in someone’s life, knowing that I have been able to be the support they need or find the support they need and I come away feeling really good about that, even though it was a sad time*“(interview No. 12). 

Although helping people manage their emotions and providing emotional support is rewarding for chaplains, it shouldalso be pointed out that being attentive to the needs of people who are in pain can threaten chaplains’ own health.

The findings of this study are in agreement with a few studies on hospital chaplaincy in certain ways. For example, Taylor et al. (12), Flannelly et al. (13) and crossly(14) discovered high levels of job satisfaction among the chaplains in their quantitative research. Similar to these studies, the present research reveals a direct relationship between occupational hazards such as burnout and workload in the sense that the more hours chaplains spend working, the higher the chances of becoming stressed and burnt out. 

Similar to what crossly(14) uncovered, chaplains of this study paid special attention to their spiritual life and their relationship with God. Not only was their spiritual and religious life an important source of satisfaction, but it was also an essential part of the reasons they chose this line of work. 

In contrast to previous studies, however, this research explores multiple resources, strategies and supportive means that chaplains may rely on in order to manage their emotions and perform their job. Generally speaking, quantitative studies often fail to consider the important factors that lead chaplains towards higher job performance. Also, previous studies have not made it clear in what ways chaplain’s health can be improved by different strategies of emotion management. 

## Conclusion

Among the chaplains who were interviewed for this study, there seems to be a high level of job satisfaction. Health conditions and quality of life also seem to be good for the most part, except for a few full-time chaplains. As already discussed, one important reason chaplains work part-time is to protect their health and avoid occupational hazards such as burnout and fatigue. Therefore, hospital chaplaincy can be a very intense and difficult job, not because of the enormity of pain and suffering chaplains see in their everyday interactions with people (far from it, the job is very rich in terms of emotional experience), but because of the workload to which some full-time chaplains are committed. There does seem to be a difference in the health condition of full-time and part-time chaplains. Quite a few full-time chaplains seemed exhausted and close to burnout. However, instead of the emotional difficulties of the job, it is rather the huge workload from which the above chaplains suffer. All chaplains, even those overloaded with work, said they love their job. It is 40 hours of intense weekly work in one of only two trauma centers in Toronto that is overwhelming.

***Limitations of the Study***

As a limitation, it should be noted that even though the small sample of 21 respondents allowed for greater depth and more meaningful interaction with respondents, the findings of this study cannot be generalized to all chaplains who work in Canada. Also, the sample is likely to be biased in terms of gender, as it was not possible to interview more than three male chaplains, in contrast to 18 females. 

## References

[1] Halpern J (2001). From detached concern to empathy: humanizing medical practice.

[2] May W (1983).

[3] Koenig HG, Pargament KI, Nielsen J (1998). Religious Coping and Health Status in Medically Hospitalized Older Adults. J Nerv Ment Dis.

[4] Pargament K Koenig HG, Perez LM (2000). The Many Methods of Religious Coping: Development and Initial Validation of RCOPE. J Clin Psychol.

[5] Clarke, P (2009). Religion, Spirituality, and Health: An Institutional Approach. The Oxford Handbook of the Sociology of Religion.

[6] Cadge W, Freese J, Christakis NA (2008). The provision of hospital chaplaincy in the United States: a national overview. South Med J.

[7] Rodrigues B, Rodrigues D, Casey D L (2000).

[8] Flannelly KJ, Weaver AJ, Handzo GF, Smith WJ (2005). J Pastoral Care Counsel.

[9] Sakurai M (2005). Ministry of Presence: Naming What Chaplains Do at theBedside.

[10] Turner J, Stets J (2006). Emotions and Health. Handbook of the Sociology of Emotions.

[11] Thoits P (1996). Managing the Emotions of Others. Symbolic Interaction.

[12] Taylor BE, Flannelly KJ, Weaver AJ, Zucker DJ (2006). Compassion Fatigue and Burnout among Rabbis Working as Chaplains. J Pastoral Care Counsel.

[13] Flannelly K Roberts SB, Weaver AJ. J Pastoral Care Counsel.

[14] CrossleyK (2002). Professional satisfaction among U.S. healthcare chaplains. J Pastoral Care Counsel.

[15] Hochschild A (1979). Emotion Work, Feeling Rules and Social Structure. Am J Sociol.

[16] Glaser B, Strauss A (1967). The Discovery of Grounded Theory.

[17] Weiss R (1994). Learning from Strangers: The Art and Method of Qualitative Interview Studies.

[18] Strauss A, Corbin J (1990). Basics of Qualitative Research: Grounded Theory Procedures and Techniques.

[19] Patton M (2001). Qualitative Research and Evaluation Methods.

[20] Lofland J, Lofland L (1995). Analysing Social Settings: A Guide to Qualitative Observation and Analysis.

[21] Miles MB, Huberman M (1994). Qualitative Data Analysis: An Expanded Sourcebook.

[22] Sandelowski M (2000). Focus on Research Methods: Whatever Happened to Qualitative Description?. Research in Nursing and Health.

[23] Bell B (2003). The Rites of Passage and Outdoor Education: Critical Concerns for Effective Programming. J Exper Edu.

[24] Denzin N (1984). On Understanding Emotion.

